# Cannabidiol-induced cellular and matrix-associated responses in primary equine sarcoid cells

**DOI:** 10.1093/jvimsj/aalaf015

**Published:** 2026-01-21

**Authors:** Ewelina Semik-Gurgul, Ewa Ocłoń, Joanna Zubel-Łojek, Rafał Pędziwiatr, Klaudia Pawlina-Tyszko

**Affiliations:** Department of Animal Molecular Biology, National Research Institute of Animal Production, Krakowska 1 Street, 32-083 Balice, Poland; Department of Infectious Diseases and Public Health Protection, University of Agriculture in Krakow, 1C Redzina Street, 30-248 Krakow, Poland; Laboratory of Recombinant Proteins Production, Faculty of Veterinary Medicine University of Agriculture in Krakow, 1C Redzina Street, 30-248 Krakow, Poland; Department of Animal Physiology and Endocrinology, University of Agriculture in Krakow, Mickiewicza 24/28 Street, 30-059 Krakow, Poland; Department of Diagnostics and Clinical Sciences, Faculty of Veterinary Medicine, University of Agriculture in Krakow, Mickiewicza 24/28 Street, 30-059 Kraków, Poland; Equine Vet Clinic EQUI-VET, Proszowice 55A, 32-100 Stogniowice, Poland; Department of Animal Molecular Biology, National Research Institute of Animal Production, Krakowska 1 Street, 32-083 Balice, Poland

**Keywords:** cannabidiol, CBD, equine sarcoid cells, matrix metalloproteinases, MMPs

## Abstract

**Background:**

Sarcoids are locally invasive skin tumors in equids, associated with bovine papillomavirus.

**Hypothesis/Objectives:**

Address potential applications of cannabidiol (CBD) in veterinary medicine. We evaluated the response of equine sarcoid cells to CBD in vitro, focusing on viability, invasiveness, and matrix remodeling.

**Animals:**

Three primary sarcoid cell lines.

**Methods:**

Cells were treated with CBD (20, 6.75, 2.25, 0.75 μM) and incubated for 6, 24, 48, 72 hours. Cell viability, cytotoxicity, and apoptosis were assessed using the ApoTox-Glo Assay. Based on these results, further analyses were performed for selected conditions only, including the assessment of cell invasiveness using the ECMatrix™ Cell Invasion Assay and the quantification of matrix metalloproteinase (MMP)-1, -2, and -9 in the culture medium by ELISA.

**Results:**

Treatment with CBD affected cell viability, cytotoxicity, and apoptosis. At 48 hours, apoptosis (measured as caspase 3/7 activity) reached 49.5% and further increased to 75% at 72 hours. Marked cytotoxicity (>96%) and decreased viability were observed at 72 hours. Cannabidiol also significantly decreased MMP-1 concentration by 48.9% at 24 hours and MMP-2 concentrations by 84% at 6 hours. Concentrations of MMP-9 also decreased by 37.2% and 45.3% at 6 and 48 hours, respectively, after treatment with 20 μM. Despite observed decreases in cell invasiveness ranging from 34% to 59% after 24 hours, these changes were not significant.

**Conclusions and clinical importance:**

Our findings support further investigation of CBD’s role in extracellular matrix modulation in sarcoid tumors.

## Introduction

Equine sarcoids represent an important health issue in the horse population, being the most common skin tumor in these animals.^1^^,^^2^ These lesions are classified as locally invasive skin growths without the ability to metastasize. In some cases, they may spread into the layers beneath the skin. They are considered biphasic tumors, developing from the growth of two different components: dermal fibroblasts and epidermal keratinocytes.^3^ Because of their structure, sarcoids are susceptible to infection and mechanical damage, potentially causing discomfort to the horse and hindering function.^4^ The causes of this fibroblastic neoplasm are multifactorial and require the simultaneous presence of several factors, including bovine papillomavirus (BPV1, BPV2, or BPV13) infection, genetic predisposition, and environmental factors such as chronic physical trauma and altered wound healing.^2^^,^^5^^,^^6^ Despite many years of research, many aspects of sarcoid pathogenesis, diagnosis, and treatment remain subject to intensive study. The ambiguous manifestations and unpredictable progression of sarcoids present substantial challenges in anticipating clinical outcomes and treatment responses for veterinarians and researchers.

The medical applications of *Cannabis* have become an important topic of public debate and academic discussion in recent years. Several experiments conducted on cell cultures and animal models have determined that cannabinoids are capable of inhibiting the proliferation of cancer cells, activating the apoptosis and autophagy process, limiting angiogenesis, and decreasing metastasis.^7–9^ Cannabidiol (CBD), a non-psychoactive cannabinoid, has been reported to inhibit the growth of several tumors, both in vitro and in vivo*.*^10–14^ Although CBD has been found to be effective against various tumors, its molecular mechanism of action remains incompletely characterized. Cannabidiol exerts cytotoxic effects on gliomas and impedes tumor cell migration in vitro*,*^15–17^ induces cell death in human leukemia cell lines^18^ and inhibits breast cancer growth.^14^ Additionally, when combined with tetrahydrocannabinol, CBD induces programmed cell death in glioma cells.^19^

Currently, no information is available on the impact of CBD on the metabolism of equine sarcoid cells, its potential to induce apoptosis, or its ability to inhibit the proliferation of sarcoid cells. In our in vitro study, we examined the effects of CBD on the viability and invasiveness of equine sarcoid cells, assessing invasiveness by both matrix metalloproteinase (MMP-1, MMP-2, and MMP-9) secretion and an invasion assay, to better characterize CBD-induced cellular responses relevant to matrix-related activity in this tumor model.

## Materials and methods

### Graphic abstract

**Figure 1:**
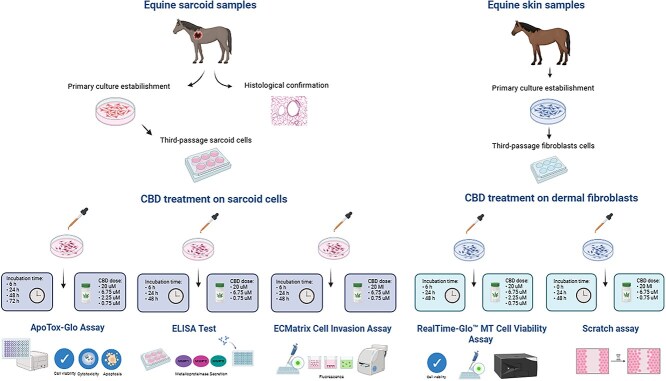
Graphical abstract illustrating the experimental design.

Figure 1 provides a visual overview of the experimental design of the study, (created using https://BioRender.com).

### Sample collection and establishment of primary sarcoid cultures

The material used to establish primary sarcoid cell cultures consisted of three equine sarcoid tissue fragments collected during standard tumor excision procedures. The tumors were histologically confirmed as described previously.^20^ In addition, the presence of BPV1 and BPV2 DNA was verified in both the tumor tissue and the corresponding cell cultures using qPCR (Table S1A and B), with healthy skin tissue serving as a negative control, as previously described.^20^^,^^21^ The tissue fragments utilized for establishing the primary sarcoid cell cultures were explanted immediately after excision and processed without prior cryopreservation. To decrease peripheral contamination, fragments collected from the central region of resected primary tumors, were placed in a Dulbecco’s Modified Eagle’s Medium with GlutaMAX (DMEM, 4500 mg/L, Gibco–Thermo Fisher Scientific, United States) supplemented with 10% fetal bovine serum (FBS, Gibco, Thermo Fisher Scientific, United States) and 1% Gibco™ Antibiotic-Antimycotic (10 000 units/mL of penicillin, 10 000 μg/mL of streptomycin, and 25 μg/mL of Gibco Amphotericin B, Thermo Fisher Scientific, United States). Upon arrival in the laboratory, typically within 1-2 hours after-excision, samples were aseptically sectioned into fragments approximately 3 × 3 × 3 mm. The fragments then were subjected to two successive washes in phosphate-buffered saline (PBS, pH 7.3-7.4), each lasting 2-3 minutes, followed by an additional rinse in DMEM GlutaMAX medium. Prepared tissue fragments were subsequently transferred to 6-well culture plates (2-3 fragments per well) and incubated under standard conditions at 37°C in a humidified atmosphere containing 5% CO₂. Cellular outgrowth from tissue explants was monitored daily after initiation of culture. Primary cell migration was first observed between days 6 and 10, with a monolayer of adherent cells becoming visible between days 12 and 14. Consistent cell growth was observed surrounding the tissue fragments once the cells reached 70%-80% confluency. At this stage, the medium was carefully aspirated, and the cell monolayer was gently washed three times with PBS to prepare for detachment. Cells then were detached by trypsinization (Tripsin-EDTA, 0.05%, Gibco, Thermo Fisher Scientific, United States), harvested, and subsequently cultured until confluence was reached in the first passage. Afterward, cells were trypsinized again and cryopreserved in CTS™ Synth-a-Freeze™ Medium (Gibco, Thermo Fisher Scientific, United States). All experimental procedures were performed on cells at the third passage.

### Equine sarcoid cell treatment and medium collection

Third-passage sarcoid cells were cultured in 6-well plates (Thermo Fisher Scientific, United States) using DMEM with GlutaMAX, supplemented with 10% FBS and 1% antibiotic-antimycotic solution, as described in section 2.2. Cultures were maintained under standard conditions until reaching 80% confluency. Upon reaching target confluency, cells were treated with CBD (NMID512B-25MG, MERCK, United States) at concentrations of 20.0, 6.75, 2.25, and 0.75 μM for incubation periods of 6, 24, 48, or 72 hours. The CBD initially was dissolved in dimethyl sulfoxide (DMSO) and subsequently diluted to the desired final concentrations in DMEM with GlutaMAX, ensuring a final DMSO concentration of 0.1% in all treatment media. The vehicle control consisted of 0.1% DMSO in DMEM with GlutaMAX.

All experiments were performed in triplicate for each of the three independent sarcoid cell lines.

### ApoTox-Glo test (viability, cytotoxicity, and apoptosis)

Cell viability, apoptosis, and cytotoxicity of CBD were assessed using the ApoTox-Glo™ Triplex Assay (Promega, United States), following the manufacturer’s protocol. Treated cells were seeded in white-walled 96-well plates at a density of 15 000 cells per well and allowed to adhere overnight. After attachment, cells were treated with CBD at concentrations ranging from 0.75 to 20.0 μM. For viability and cytotoxicity assessments, live-cell protease activity was measured using a cell-permeant peptide substrate (GF-AFC), which is cleaved only by intact, viable cells to produce a fluorescent signal. Dead-cell protease activity was measured using a cell-impermeant peptide substrate (bis-AAF-R110), which emits a separate fluorescent signal when cleaved by proteases released from cells with compromised membranes. Fluorescence measurements were taken at 6, 24, 48, and 72 hours using a TECAN Infinite M200 PRO microplate reader, with data reported as fluorescence intensity relative to control cells.

Apoptosis was quantified by measuring caspase-3/7 activity using the Caspase-Glo® 3/7 Reagent included in the ApoTox-Glo Assay. Caspase-3/7 activity was assessed by adding the Caspase-Glo® 3/7 Reagent to each well in a 1:1 ratio with the culture medium. The plate then was shaken gently at room temperature for 30 minutes, and luminescence was measured to assess caspase activation. As a positive control, cells were treated with 5 μM staurosporine (0.1% final DMSO; Merck, United States). Luminescence intensity was determined using a TECAN Infinite M200 PRO microplate reader. Luminescence was measured at 6, 24, 48, and 72 hours after the addition of CBD.

### ELISA test

The concentrations of human MMP-2 (inter-assay coefficient of variation [CV] < 12%; intra-assay CV < 10%; MERCK, United States), equine MMP-1 (inter-assay CV < 15%; intra-assay CV < 10%; St John’s Laboratory, United States), and equine MMP-9 (inter-assay CV < 12%; intra-assay CV < 10%; Invitrogen, Thermo Fisher Scientific, United States) proteins in cell culture supernatants exposed to CBD (0.75, 6.75, and 20.0 μM concentration) for 6, 24, or 48 hours were measured using ELISA following the manufacturers’ instructions. An MMP-2 ELISA kit designed for use in humans was employed, because full-length human and equine MMP-2 proteins share approximately 96% sequence homology, including approximately 97% identity within the catalytic domain, which supports antibody cross-reactivity.

### ECMatrix cell invasion assay

The effect of CBD on sarcoid cell invasion was assessed using the QCM 24-well Cell Invasion Assay kit (MERCK, United States), which includes polycarbonate membrane inserts (8 μM pore size) coated with a thin layer of ECMatrix. The basement membrane layer was rehydrated by adding 300 μL of warm, serum-free medium to the inserts and incubating them for 30 minutes in a cell culture incubator. A suspension of sarcoid cells (0.5 × 10^6^ cells per ml) in serum-free medium supplemented with CBD (0.75, 6.75, and 20.0 μM) was seeded onto ECMatrix-coated transwells in invasion chambers. After 6, 24, and 48 hours of incubation, the medium in the membrane chamber was transferred to a new harvesting tray containing 225 μL of detachment solution and incubated for 30 minutes at 37°C. Cells were completely dislodged from the underside of the membrane by gently tilting the membrane multiple times. Subsequently, 75 μL of lysis buffer/dye solution was added to each sample, followed by incubation for 15 minutes at room temperature. Fluorescence measurements then were taken using a TECAN Infinite M200 PRO microplate reader at 480 nm/520 nm.

In addition, at each CBD concentration (0.75, 6.75, and 20.0 μM) and time point (6, 24, and 48 hours), ECMatrix and any remaining cells in the upper chamber were removed using cotton swabs. Cells that had invaded to the lower surface of the membrane were fixed in 4% paraformaldehyde and stained with 0.1% crystal violet. Cells in five microscopic fields (at 200× magnification) were counted and photographed.

### Reference analysis of CBD response in normal equine dermal fibroblasts

To assess whether the cellular responses to CBD observed in equine sarcoid cells were tumor-specific or reflected broader cytotoxic effects, a reference experiment was conducted using primary normal equine dermal fibroblasts. The aim was to determine whether CBD alters viability or behavior in non-neoplastic dermal cells under the same experimental conditions. For this purpose, we examined cell viability as a measure of metabolic activity and cell migration as an indicator of coordinated movement. Although the scope was deliberately limited, these two readouts were deemed sufficient to identify potential nonspecific effects of CBD on healthy dermal fibroblasts. Primary fibroblast cultures were established from fresh skin fragments obtained from a healthy cold-blooded horse at the slaughterhouse. Tissue processing, explant culture, and expansion were carried out as described for sarcoid cells (Section 2.2). All experiments were performed using cells at passage 3.

Cell viability was assessed using the RealTime-Glo™ MT Cell Viability Assay (Promega), which detects the reducing potential of metabolically active cells using a luminescent signal. Fibroblasts were treated with CBD at concentrations of 20, 6.75, 2.25, and 0.75 μM, or 0.1% DMSO (vehicle control), and incubated for 6, 24, and 48 hours. Cells were seeded in 96-well white-walled plates, and luminescence was recorded using a TECAN Infinite M200 PRO microplate reader. Values were normalized to the control (0.1% DMSO).

Cell migration was evaluated using a scratch assay performed in 12-well plates. Confluent fibroblast monolayers were wounded using a sterile pipette tip, and detached cells were removed by washing. Fresh medium containing CBD at concentrations of 20, 6.75, or 0.75 μM, or 0.1% DMSO, was applied. Images were taken at 0, 24, and 48 hours. Each condition was tested in triplicate wells. The percentage of wound closure was quantified using ImageJ software.^22^

### Statistical analysis

All results are presented as the mean ± SD or error for technical triplicate of the three independent sarcoid cell lines. The statistical analyses were conducted using JASP software version 0.16.3 (JASP Team, 2022). A two-way analysis of variance (ANOVA) was utilized to assess the effects of CBD dose and exposure time on the measured variables. Post hoc comparisons were performed using Tukey’s test to identify significant differences between groups. Before the analysis, the assumption of homogeneity of variance was assessed using Levene’s test. In cases when the assumption was violated, a natural logarithmic transformation of the data was performed to equalize the variances. For the reference analysis in normal dermal fibroblasts, viability data were analyzed using one-way ANOVA with Tukey’s post hoc test, whereas wound closure data from the scratch assay were evaluated using the non-parametric Mann–Whitney U test.

## Results

### Effect of CBD on cell viability, cytotoxicity and apoptosis

**Figure 2:**
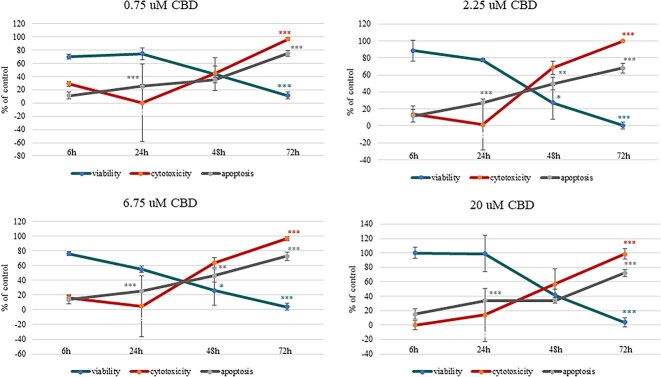
Impact of CBD concentrations (20, 6.75, 2.25, and 0.75 μM) and treatment durations (6, 24, 48, and 72 hours) on viability, cytotoxicity, and apoptosis in equine sarcoid cells. Results are shown as mean ± SE (standard error) of three technical replicates for each of three independent sarcoid cell lines. ^*^P < .05; ^**^P < .01; ^***^P < .001 compared to control cells as determined by the ANOVA test.

The ApoTox-Glo assay was used to simultaneously measure cell viability, cytotoxicity, and apoptosis in CBD-treated cells under specific experimental conditions. All measurements were compared to control cells set at 100% viability. An ANOVA test identified significant effects of exposure time (*P* < .001) and CBD dose (*P* < .001), as well as a significant interaction between time and dose (*P* < .001) on cell viability, cytotoxicity, and apoptosis. However, post hoc comparisons identified no significant differences in cell viability and cytotoxicity between CBD-treated groups and the control group (0.1% DMSO) at any concentration or time point, except at 72 hours (*P* > .05). At 72 hours, all CBD concentrations induced a dramatic decrease in cell viability, with the 2.25 μM dose resulting in only 0.4% viability (*P* < .001; Figure 2, Table S2). This dose also caused substantial cytotoxicity (96.7%-98.6%), with significant p-values (*P* < .001 for all exposure times; Figure 2, Table S2). Therefore, all subsequent experiments were conducted without including the 72-hour time point. Furthermore, the 2.25 μM CBD dose also was excluded from additional evaluations, because it exhibited effects comparable to those seen with the 0.75 and 6.75 μM doses.

Apoptosis, as measured by caspase 3/7 activity, was found to be both dose-dependent and time-dependent. After 6 hours of incubation with CBD, no significant increase was found in the number of apoptotic cells. After 24 hours, CBD significantly activated cellular apoptosis, with rates reaching 34.2% (*P* < .001) at a concentration of 20 μM and 25.2% (*P* < .001) at 6.75 μM. At the 48-hour time point, caspase 3/7 activation continued, increasing to approximately 34.2%-49.5% (*P* < .01). At the 72-hour time point, the activation of caspase-3/7 in apoptotic cells peaked, with the 0.75 μM dose reaching 75% apoptotic cells (*P* < .001; Figure 2, Table S2).

### Effect of CBD on selected metalloproteinase secretions

**Figure 3:**
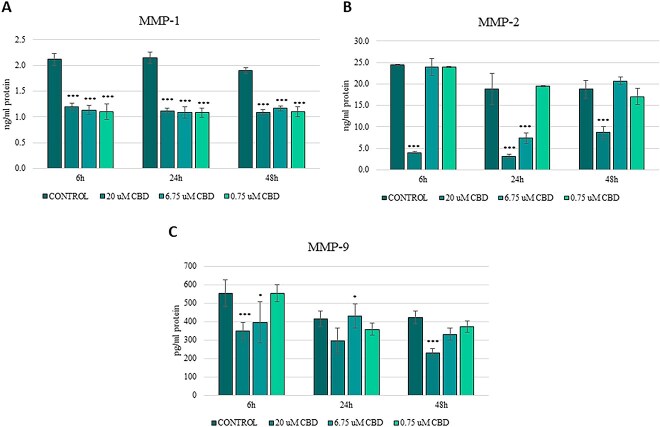
Levels of MMPs in cell culture medium under different CBD concentrations (20, 6.75, and 0.75 μM) and incubation times (6, 24, and 48 hours). Results are shown as mean ± SD (standard deviation) of three technical replicates for each of three independent sarcoid cell lines. ^*^P < .05; ^**^P < .01; ^***^P < .001 compared to control cells as determined by the ANOVA test.

The effect of CBD on the secretion of MMP-1, MMP-2, and MMP-9 proteins was assessed using the ELISA method. A significant effect of CBD dosage (*P* < .001) on the secretion of MMP-1 was observed in the equine sarcoid cells. A significant decrease was observed across all CBD concentrations after 24 hours of incubation (*P* < .001). Control cells reached 2.14 ± 0.26 ng/mL, whereas CBD-treated groups showed MMP-1 concentrations between 1.08 ± 0.11 and 1.11 ± 0.13 ng/mL, representing an average decrease of approximately 49% (Figure 3A, Table S4).

In the case of MMP-2, the most pronounced inhibition was observed at 6 hours after treatment with 20 μM CBD (*P* < .001), decreasing secretion from 24.41 ± 2.93 ng/mL to 3.98 ± 0.49 ng/mL. At this time point, neither 6.75 μM nor 0.75 μM resulted in significant changes. At 24 hours, 20 μM again led to a marked decrease (3.08 ± 0.37 ng/mL, *P* < .001), whereas 6.75 μM significantly decreased concentrations to 7.33 ± 0.89 ng/mL (*P* < .01; Figure 3B, Table S4).

At 6 hours, 20 μM CBD decreased MMP-9 concentrations from 554.73 ± 66.57 pg/mL to 348.08 ± 41.79 pg/mL, indicating a 37% decrease (*P* < .001). The 6.75 μM concentration also caused a significant decrease to 396.12 ± 47.53 pg/mL (*P* < .05). After 48 hours, 20 μM caused a further significant decrease in MMP-9 concentration to 296.88 ± 35.63 pg/mL (*P* < .001), whereas moderate and low doses resulted in smaller, non-significant changes (Figure 3C, Table S4).

### Effect of CBD on cell invasion/migration ability

**Figure 4:**
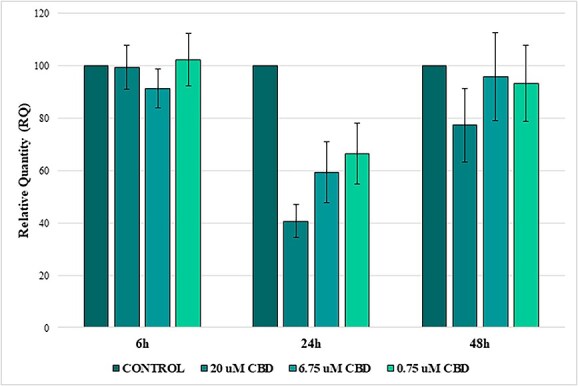
Effects of CBD concentrations (20, 6.75, and 0.75 μM) on sarcoid cell invasion across time points (6, 24, and 48 hours). Quantification of invading cells treated with CBD, expressed relative to those treated with DMSO. Results are shown as mean ± SD of three technical replicates for each of three independent sarcoid cell lines. ^*^P < .05; ^**^P < .01; ^***^P < .001 compared to control cells as determined by the ANOVA test.

We also examined the effect of CBD on the invasiveness of sarcoid cells. All measurements were compared against the control cells, which were set at 100%. Analysis of variance determined that incubation time with CBD (*P* < .001) was a more significant factor influencing cell invasiveness than its concentration (Table S3). The results indicated that after 24 hours, all three concentrations of CBD resulted in decreased cell invasiveness compared with the control cells, with the 20 μM dose showing the most pronounced effect (approximately 59% decrease). However, this decrease did not reach significance (*P* = .07). The 6.75 μM and 0.75 μM doses resulted in decreases in invasiveness of approximately 40.6% and 33.6%, respectively (*P* > .05). After 48 hours, the 20 μM dose resulted in a 22.7% decrease in invasiveness (*P* = .99), whereas other concentrations showed marginal effects, all of which were non-significant (*P* > .05; Figure 4, Figure S1 and Table S3).

### Effect of CBD on viability and migration of normal equine dermal fibroblasts

**Figure 5:**
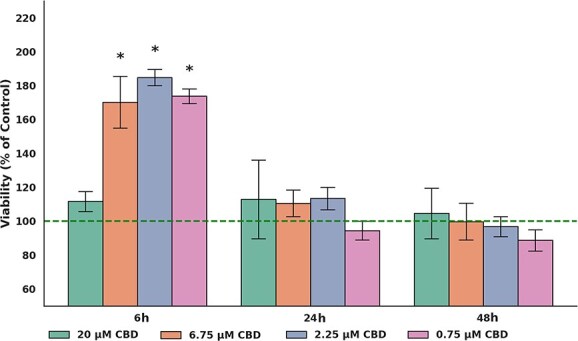
Effects of CBD on the viability of normal equine dermal fibroblasts. Viability of normal equine dermal fibroblasts exposed to CBD at concentrations of 0.75, 2.25, 6.75, and 20 μM was assessed at 6, 24, and 48 hours using the RealTime-Glo™ MT Cell Viability Assay. Results are expressed as a percentage of the respective control group, indicated by the horizontal reference line (100%). Data represent mean ± SEM from six biological replicates. Statistical analysis was performed using one-way ANOVA followed by Tukey’s post hoc test. Asterisks indicate significant differences vs. control (^*^P < .05).

In normal equine dermal fibroblasts, viability remained stable or slightly increased after CBD exposure. At 6 hours, cells treated with 6.75, 2.25, and 0.75 μM exhibited significantly higher viability compared with the control group (all *P* < .05), whereas 20 μM had no effect. At 24 and 48 hours, no significant differences in viability were observed between any CBD-treated group and the control (all *P* > .05). Overall, CBD did not decrease fibroblast viability at any dose or time point (Figure 5, Table S5A).

**Figure 6:**
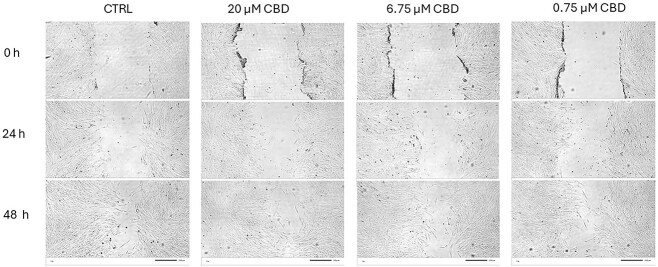
Effects of CBD on migration of equine normal fibroblasts in a scratch assay. Representative phase contrast microscopy images of equine normal skin fibroblasts in a wound healing (scratch) assay aftertreatment with CBD at concentrations of 0.75, 6.75, and 20.0 μM. The control condition consisted of 0.1% DMSO (vehicle control). Images were taken at 0, 24, and 48 hours after scratch formation to qualitatively observe cell migration. Each condition was performed in triplicate. No significant differences in wound closure were observed between the groups. Scale bar = 200 μM.

In the scratch assay, wound closure at 24 hours was comparable between CBD-treated fibroblasts and controls. At 48 hours, cells exposed to 6.75 and 20 μM had higher closure values than the control, although the difference was not significant (Figure 6, Figure S2 and Table S5B).

## Discussion

Cannabinoids have been extensively studied for their various biological effects in human cell models, but their potential applications in veterinary oncology have not been thoroughly examined. In particular, research on the impact of CBD on tumor cells of animal origin is limited, emphasizing the need for additional studies using in vitro models in species of veterinary interest. In our study, we first investigated whether CBD has specific effects on equine sarcoid cells, measuring its impact on cell viability, apoptosis, and cytotoxicity. Our analyses indicated a significant impact of CBD dose and exposure time on cell viability, apoptosis, and cytotoxicity. Although not all comparisons between groups were significant, our results demonstrate a dose- and time-dependent decrease in cell viability, with significant decreases occurring at higher concentrations and prolonged exposure to CBD. These findings are consistent with previous studies,^23–25^ that showed that CBD decreases the viability of various cancer cell lines, including human colorectal cancer cells and gingival squamous cell carcinoma cells (OECM-1). Similar to our data, more pronounced effects were reported at higher concentrations. The observed cytotoxicity was minimal at 6 hours, but a notable increase was observed at the lowest concentration (0.75 μM), suggesting that cell death mechanisms at this early stage may involve mild cellular damage that does not yet substantially decrease viability. Mechanisms such as necrosis or autophagy may have been at play during this period, warranting further investigation. These findings are consistent with results from other studies,^10^^,^^23^^,^^26^^,^^27^ supporting the hypothesis that CBD induces cell death through multiple pathways. Interestingly, no significant cytotoxicity was observed at 6-24 hours, potentially indicating cellular adaptation, activation of repair mechanisms, or transition to a more orderly form of cell death, such as apoptosis. The decrease in cytotoxicity at this time also could be related to activation of repair mechanisms or a transient compensatory effect in the cell population. Additionally, the lack of significant activation of caspase-3/7 at the early stage (6 hours) suggests that the cells may undergo delayed apoptosis. The time-dependent peak in apoptosis, with caspase-3/7 activity reaching 75% of cells at 0.75 μM after 72 hours, emphasizes apoptosis as the dominant mechanism of cell death after prolonged exposure. In agreement with our study, previous studies reported that treatment with CBD induces cytotoxicity in a time-dependent manner by selectively decreasing cell viability and inducing apoptosis in ovarian carcinoma cells^28^ or in human and canine glioma cells.^29^ The concentration of CBD also plays a crucial role in its cytotoxicity. The obtained results show a cytotoxic effect at the lowest dose (0.75 μM) after 6 hours, which is less common in other studies where higher concentrations (eg, 5-20 μM) are typically required to observe significant early effects.^28^^,^^30^^,^^31^ This finding suggests that equine sarcoid cells may be susceptible to CBD, perhaps because of unique metabolic or signaling pathways in these cells. Although high concentrations of CBD (eg, 20 μM) significantly activated apoptosis in equine sarcoid cells after 24 hours, cytotoxicity itself was not significant at this time point. This delayed cytotoxicity at higher doses contrasts with many studies in cancer cells, where high CBD concentrations (10-25 μM) frequently induce rapid and robust cytotoxic effects within the first 24 hours. For example, studies on MCF-7 breast cancer cells^30^ and glioma cells^15^ reported significant cytotoxicity at concentrations exceeding 10 μM within 24 hours, attributed to mechanisms such as mitochondrial dysfunction and oxidative stress. In contrast, our data suggest that equine sarcoid cells might exhibit an adaptive response to higher doses during the early phase, delaying overt cytotoxic effects until 48-72 hours, when significant cell death occurs across all tested concentrations.

Our results indicate that CBD significantly modulates the secretion of MMP-1, MMP-2, and MMP-9 in equine sarcoid cells. These matrix metalloproteinases are central to extracellular matrix (ECM) degradation and tumor cell invasion, and their dysregulation is closely linked to enhanced tissue infiltration and local aggressiveness. In equine sarcoids, MMPs are known to be upregulated and associated with ECM remodeling and tumor progression, particularly in the context of bovine papillomavirus involvement.^32–38^ Matrix metalloproteinase-1, expressed in neoplastic sarcoid cells, is implicated in local tumor aggressiveness and has been shown to be upregulated by BPV-1 activity.^33^^,^^36^ In our study, CBD significantly decreased MMP-1 concentrations across all tested CBD concentrations and time points, with the most pronounced effect (approximately 48.9%) observed at 24 hours. Although limited evidence is available on CBD’s effect on MMP-1 in cancer cells, a biphasic response has been described in non-tumor models such as human gingival fibroblasts, where low CBD concentrations increased, and higher doses decreased MMP-1 concentrations.^39^ This finding suggests that CBD’s effects on MMP-1 are dose-dependent and highly context-specific. Our findings indicate a consistent inhibitory action of CBD on MMP-1 in sarcoid cells, supporting its relevance as a regulatory target in tumor-associated ECM degradation. Cannabidiol also decreased the concentrations of MMP-2 and MMP-9, two proteolytic enzymes strongly implicated in tumor cell invasion and motility. Our data indicated robust early inhibition of both proteins at higher CBD concentrations, with some delayed effects at lower concentrations. These results are in agreement with previous studies that found MMP-2/9 suppression by CBD in glioma and triple-negative breast cancer models.^40^^,^^41^ Taken together, our findings suggest a dose- and time-dependent molecular impact of CBD on ECM remodeling in equine sarcoid cells, suggesting its potential to disrupt invasion-associated mechanisms at the biochemical level.

To further explore CBD’s influence on invasive behavior, we conducted a cell invasion assay using a reconstituted ECM membrane. The changes (34%-59% at 24 hours), however, did not reach significance. At the 24-hour time point, cell viability remained high (77.5%), and no significant apoptosis or cytotoxicity was observed, indicating that the decreased invasion was not an artifact of cell loss. These findings are consistent with results observed in other tumor models, including highly invasive bladder cancer cells. In a study of T24 bladder cancer cells, CBD at a concentration as low as 2.5 μM significantly decreased invasion, emphasizing its potential to inhibit invasive behavior even in aggressive cancer models.^42^ Similarly, several studies have shown that CBD can decrease invasion as well as migration in various cancers, including breast cancer, lung cancer, glioblastoma, and head and neck squamous cell carcinoma.^26^^,^^31^ Although equine sarcoids are not metastatic by nature, their classification as locally invasive skin tumors emphasizes the importance of targeting mechanisms driving their tissue-specific invasiveness.

Our study had several limitations that should be taken into account when interpreting our findings. One important constraint is limited sample diversity, because the study included only three primary equine sarcoid cell lines. This narrow representation may not fully capture the biological heterogeneity of sarcoid tumors and therefore could limit the generalizability of the results. Another limitation concerns the range of experimental conditions. Although the selected CBD concentrations (0.75, 6.75, and 20 μM) and exposure durations (6, 24, and 48 hours) provided valuable insights, they may not reflect the complete spectrum of potential cellular responses, particularly at lower or higher doses or with prolonged exposure.

In addition to these considerations, we sought to determine whether the effects observed in sarcoid cells were specific to neoplastic transformation. To this end, we conducted parallel analyses using primary normal equine dermal fibroblasts. Notably, CBD treatment did not decrease cell viability at any concentration or time point in these non-tumor cells. Similarly, fibroblast migration in the scratch assay proceeded comparably to that of vehicle-treated controls, with no detectable impairment in wound closure. These observations are consistent with previous findings in fibroblasts from humans, which likewise exhibited no cytotoxic response to similar CBD concentrations.^42^^,^^43^ Collectively, these data support the interpretation that the effects seen in sarcoid cells likely reflect tumor-specific responsiveness rather than non-selective cytotoxicity against equine dermal cells.

Finally, it is important to acknowledge that the use of an in vitro system inherently limits the extrapolation of results to in vivo contexts, where factors such as the tumor microenvironment and immune interactions may significantly influence cellular responses.^43^^,^^44^ In conclusion, our study provides preliminary evidence that CBD may exert anti-proliferative effects on equine sarcoid cells, as indicated by decreased cell viability and increased caspase 3/7 activity under specific experimental conditions, suggesting the involvement of apoptotic mechanisms. Additional studies are needed to confirm and extend these findings across a broader range of models and conditions. Notably, CBD also significantly suppressed the concentrations of matrix metalloproteinases MMP-1, MMP-2, and MMP-9, which are essential for ECM degradation and are closely associated with the invasive behavior of tumor cells. However, the decrease in cell invasion observed in the functional assay did not reach significance. These findings suggest that CBD may interfere with matrix-associated mechanisms relevant to tissue infiltration, even at sub-cytotoxic concentrations. Importantly, complementary analyses using normal equine dermal fibroblasts showed no decrease in viability or migration, indicating that the effects observed in sarcoid cells likely reflect tumor-associated responsiveness rather than nonspecific toxicity. Overall, equine sarcoid cells exhibit clear biological responsiveness to CBD, supporting its relevance as a modulator of matrix remodeling and invasive potential in this tumor model.

## Supplementary Material

aalaf015_Supplemental_Files

## Off-label antimicrobial declaration

Authors declare no off-label use of antimicrobials.

## Institutional animal care and use committee or other approval declaration

All procedures followed Polish law (The Polish Act on the Protection of Animals Used for Scientific or Educational Purposes of January 15, 2015, which implements Directive 2010/63/EU of the European Parliament on protecting animals used for scientific purposes).

## Abbreviations

BPV: bovine papillomaviruses
CBD: cannabidiol
ECM: extracellular matrix
ELISA: enzyme-linked immunosorbent assays
MMP: matrix metalloproteinase
qPCR: quantitative polymerase chain reaction
