# Supplementary figures and images for: Cannabidiol-induced cellular and matrix-associated responses in primary equine sarcoid cells
Source: J Vet Intern Med. 2026 Jan 21;40(1):aalaf015. doi: 10.1093/jvimsj/aalaf015 (PMC12881954; doi:10.1093/jvimsj/aalaf015)

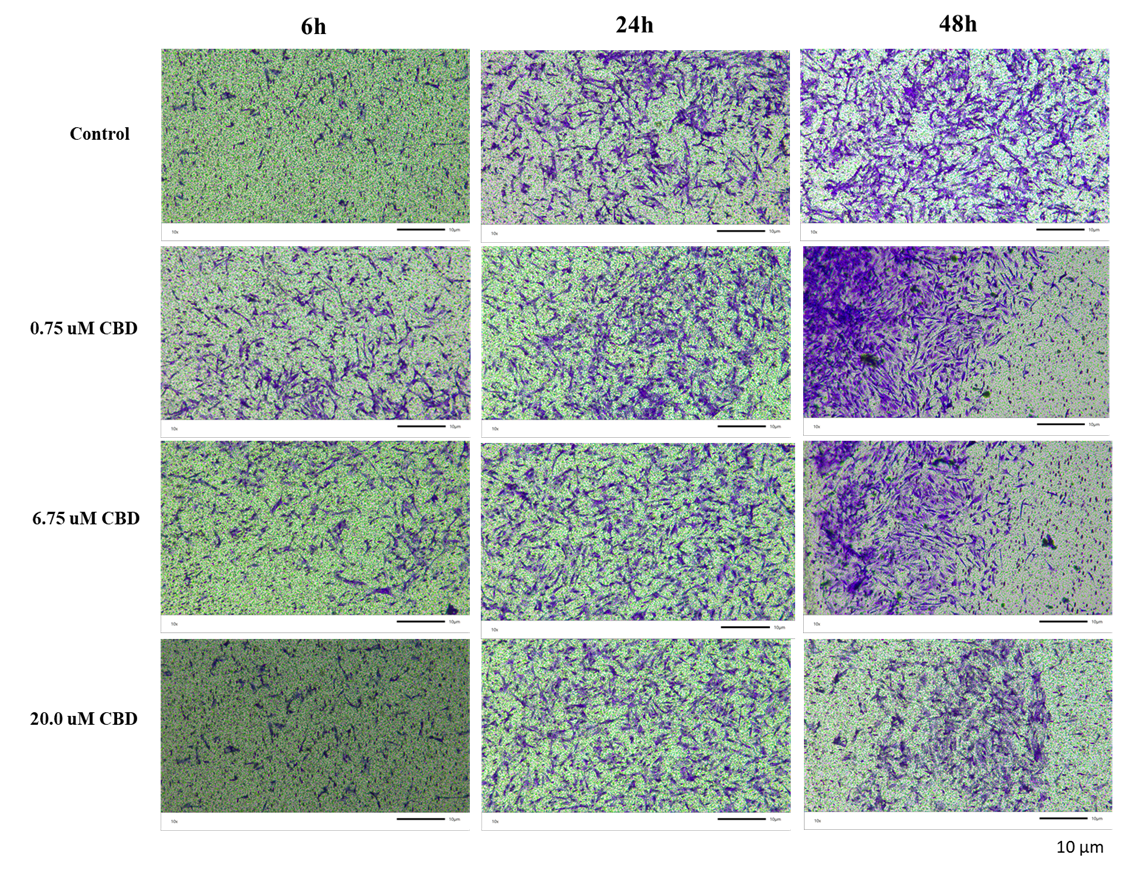

Supplement: aalaf015_Supplemental_Files [file aalaf015_supplemental_files.zip › Figure_S1_aalaf015.tif]

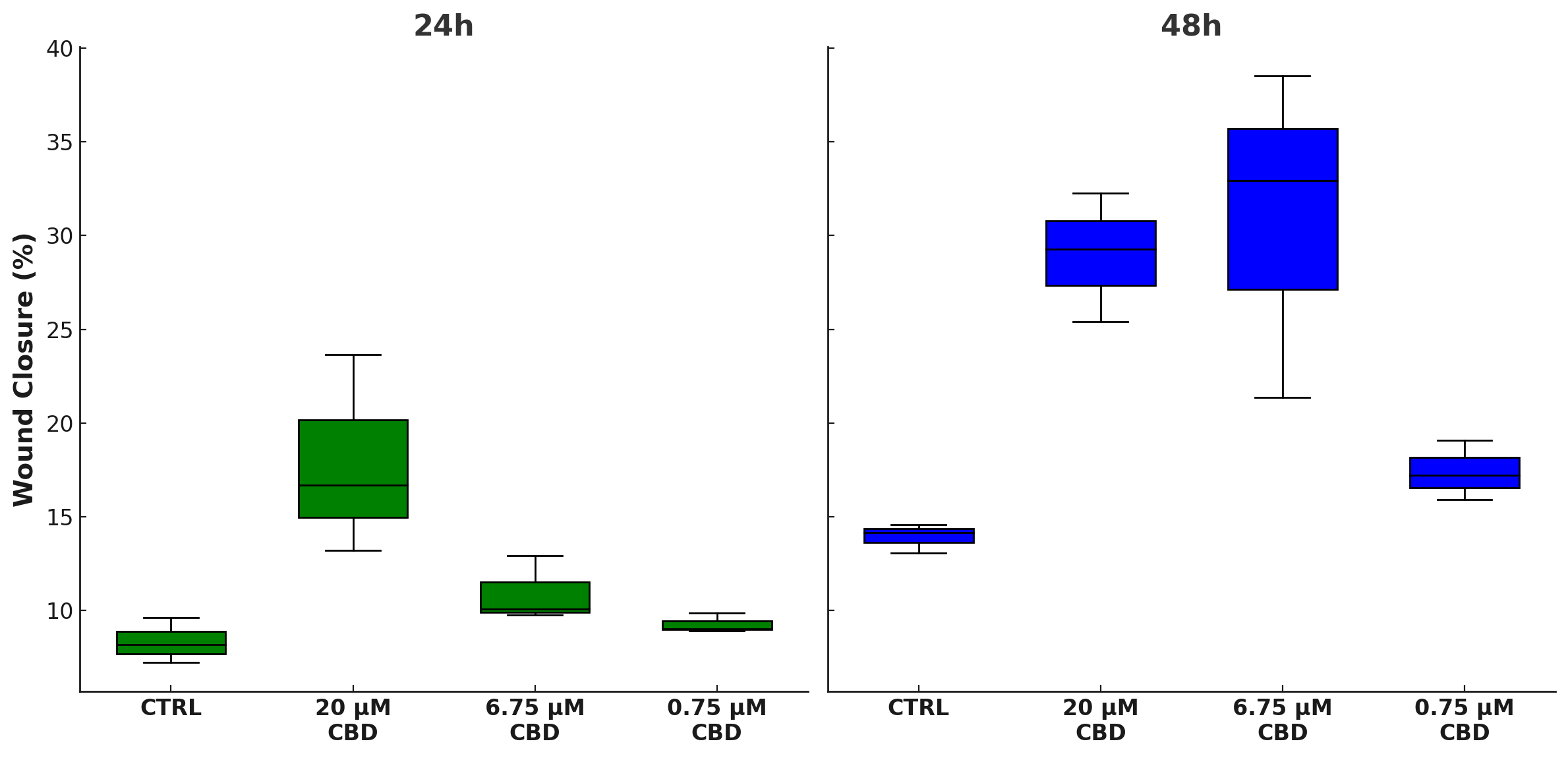

Supplement: aalaf015_Supplemental_Files [file aalaf015_supplemental_files.zip › Figure_S2_aalaf015.png]
